# Do Nutritional Supplements Have a Role in Age Macular Degeneration Prevention?

**DOI:** 10.1155/2014/901686

**Published:** 2014-01-23

**Authors:** Maria D. Pinazo-Durán, Francisco Gómez-Ulla, Luis Arias, Javier Araiz, Ricardo Casaroli-Marano, Roberto Gallego-Pinazo, Jose J. García-Medina, Maria Isabel López-Gálvez, Lucía Manzanas, Anna Salas, Miguel Zapata, Manuel Diaz-Llopis, Alfredo García-Layana

**Affiliations:** ^1^University of Valencia, Spain; ^2^The Ophthalmic Research Unit “Santiago Grisolía”, Valencia, Spain; ^3^University of Santiago de Compostela, Spain; ^4^The Institute Gomez-Ulla, Santiago de Compostela, Spain; ^5^Foundation RetinaPlus, Spain; ^6^University of Barcelona, Spain; ^7^Retina Section, Department of Ophthalmology, Bellvitge University Hospital, Barcelona, Spain; ^8^Vitreous and Retina Department, UPV/EHU and Instituto Clínico Quirúrgico de Oftalmología (ICQO), University of the Basque Country, Bilbao, Spain; ^9^Clinic Institute of Ophthalmology, Clinic Hospital of Barcelona, University of Barcelona, Barcelona, Spain; ^10^Macula Section, Department of Ophthalmology, University and Polytechnic Hospital La Fe, Valencia, Spain; ^11^University of Murcia, General University Hospital Reina Sofia, Murcia, Spain; ^12^Ophthalmic Reseach Unit “Santiago Grisolia”, Valencia, Spain; ^13^The University of Valladolid, Diabetes and Telemedicine Unit at the IOBA, Spain; ^14^The Retina Unit of the Clinic University Hospital of Valladolid, Spain; ^15^The University of Valladolid, Spain; ^16^The Vitreo-Retina Unit of the Clinic University Hospital of Valladolid, Spain; ^17^Research Institute of the Hospital of Vall Hebron, Barcelona, Spain; ^18^Retina Section of the Hospital of Vall Hebron, The Universidad Autònoma de Barcelona, Barcelona, Spain; ^19^Faculty of Medicine, University of Valencia, Valencia, Spain; ^20^University and Polytechnic Hospital La Fe, Valencia, Spain; ^21^University of Navarra Clinic, Pamplona, Spain

## Abstract

*Purpose*. To review the proposed pathogenic mechanisms of age macular degeneration (AMD), as well as the role of antioxidants (AOX) and omega-3 fatty acids (**ω**-3) supplements in AMD prevention. *Materials and Methods*. Current knowledge on the cellular/molecular mechanisms of AMD and the epidemiologic/experimental studies on the effects of AOX and **ω**-3 were addressed all together with the scientific evidence and the personal opinion of professionals involved in the Retina Group of the OFTARED (Spain). *Results*. High dietary intakes of **ω**-3 and macular pigments lutein/zeaxanthin are associated with lower risk of prevalence and incidence in AMD. The Age-Related Eye Disease study (AREDS) showed a beneficial effect of high doses of vitamins C, E, beta-carotene, and zinc/copper in reducing the rate of progression to advanced AMD in patients with intermediate AMD or with one-sided late AMD. The AREDS-2 study has shown that lutein and zeaxanthin may substitute beta-carotene because of its potential relationship with increased lung cancer incidence. *Conclusion*. Research has proved that elder people with poor diets, especially with low AOX and **ω**-3 micronutrients intake and subsequently having low plasmatic levels, are more prone to developing AMD. Micronutrient supplementation enhances antioxidant defense and healthy eyes and might prevent/retard/modify AMD.

## 1. Introduction

Age-related macular degeneration (AMD) is the most common cause of blindness in the Western world. AMD has a chronic progressive course and may require lifelong observation and therapy, becoming a socioeconomic problem as the proportion of the aged population is continuously increasing [1].

The evidence of extensive decline in quality of life and increased need of daily living assistance after long follow-up of patients with AMD substantiates the need to prevent vision loss and progression to blindness.

Important advances in the understanding of AMD pathogenesis have been focused on the role of oxidative damage into the retina [2]. It is clearly established that reactive oxygen species (ROS) and oxidized lipoproteins are pivotal sources of cell and tissue stress constructing adequate background for parainflammation in the aging retina. This chronic situation contributes to the development and/or progression of AMD [3]. Angiogenesis and its downstream effects are important milestones in AMD [4, 5]. Furthermore, increasing evidence supports the fact that OS and apoptosis are closely linked processes and that both are implicated in the pathophysiologic mechanisms of AMD [2, 4, 5].

All these were generating the foundation for further epidemiological and interventional studies dealing with the role of diet and nutritional supplements in the incidence and progression of AMD [6]. The age-related macular degeneration study (AREDS) showed that high doses of zinc and vitamins reduced the risk of vision loss and progression to late AMD and recommended their use in patients with intermediate AMD or late AMD in one eye. AREDS2 study has recently reported that the beta-carotene used in its first study must be changed to lutein and zeaxanthin in order to improve security without decreasing efficacy. However, AREDS2 was not able to demonstrate that omega-3 in a nutritional supplement further reduces the risk when used in addition to lutein and high doses of vitamins and zinc in a well-nourished population [7].

In addition, there is no data of the effect of nutritional supplements for AMD prevention when lower doses of antioxidants, vitamins, and zinc are used, as it happens in European countries. As a result, many ophthalmologists are confused of whether or not to use these agents in everyday clinical practice.

As a major cause of visual impairment-related quality-of-life in older adults, new clues on the redox status, angiogenesis, inflammation, as well as in the apoptosis versus cell survival regulation in AMD are needed. In this setting, the current review tries to arise data from epidemiological and interventional studies about the role of antioxidants and the omega-3 fatty acids in AMD prevention.

## 2. A Fresh Look at the Pathogenic Mechanisms of AMD

### 2.1. Oxidative Stress

**Figure 1:**
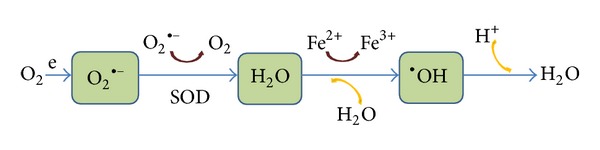
Oxidative stress (OS) is widely accepted as a key player in the initiation and progression of ocular diseases, including AMD. The chain reactions of reactive oxygen species (ROS) include the anion superoxide (O_2_^•−^), hydrogen peroxide (H_2_O_2_), and hydroxyl radical (^•^OH), all of them being able to importantly damage the cells through oxidation of lipids, proteins, and nucleic acids. These alterations lead to changes in protein function. Abbreviations: e: electron, SOD: superoxide dysmutase, and Fe: iron.

Oxidative stress (OS) results from the imbalance between the prooxidants and the antioxidant defenses leading to cellular damage and death caused by ROS [8]. The ROS are partially reduced metabolites of molecular oxygen formed through a variety of processes: normal metabolic reactions, environmental agents, or transduction pathways [9]. These ROS include oxygen free radicals, singlet oxygen, hydrogen peroxide, and their respective metabolic by products [10], as shown in Figure 1. The retina is especially vulnerable to OS because of its high polyunsaturated fatty acids (PUFAS) concentration [11–13], the elevated oxygen consumption, its exposure to light, and a wide spectrum of endogenous/exogenous injuries [14–19]. The OS role in the pathogenic mechanisms of AMD has been extensively analyzed and diverse pathogenic AMD theories have arisen. Zarbin suggested [18] that decreased oxygen delivery/metabolic “distress” may induce to the retinal pigment epithelial (RPE) cells to synthesize substances leading to choroidal neovascular growth. In this case, the RPE atrophy (probably also followed by choriocapillaris and photoreceptor atrophy), may be considered a response to decreased nutrients as well as the increased metabolic alterations in retinal areas with excessive accumulation of extracellular debris.

Macular pigment protects the macula against oxidative damage. It is mainly constituted by two dihydroxycarotenoids, lutein, and zeaxanthin, acting as an optical filter that absorbs short-wavelength visible light. Carotenoids also demonstrate antioxidant activity [20]. Eyes with a predisposition to develop AMD or which already have developed the disease have considerably less macular pigment and a greater risk of oxidative damage compared with healthy eyes [21]. In this context, clinical and experimental studies have demonstrated that photochemical macular injury is attributable to OS [21–23]. Age-related accumulation of low-molecular weight (phototoxic pro-oxidant) melanin oligomers within RPE lysosomes may contribute to a reduced photoreceptor discs phagocytosis [24]. Furthermore, the retinal pigment epithelial cell exosomes had specific signaling phosphoproteins affected by OS [25].

In this scenario, the PUFAs oxidation leads to additional ROS generation in the retina. These oxidized acids are not correctly cleaved in the lysosomes of the RPE cells and therefore are accumulated in the form of lipofuscin, which in turn is stored in Bruch's membrane as drusen. The latter can stimulate a wide variety of immune responses, including phagocytosis. In fact there is cumulative data indicating to lipofuscin as an important photoreactive agent and the result of the specific oxidative injury to the photoreceptor outer segments [26]. Docosahexaenoic acid (DHA22:6*ω*3), the principal *ω*3-PUFA in the retina, has also been reported to exert noticeable antioxidant, anti-inflammatory, and antiangiogenic effects in both “*in vivo*” and “*in vitro*” experiments [27–29].

Mitochondria are major sources of ROS, as by products of the energy metabolism. Increased ROS damages lipids, proteins, and nucleic acids. In several studies the increase in mitochondrial DNA (mtDNA) damage and mutations and the decrease in the efficacy of DNA repair have been correlated with the occurrence and the stage of AMD [30]. In fact, these authors reported that lymphocytes exhibited a higher amount of total endogenous basal and oxidative DNA damage in AMD patients. Furthermore, lymphocytes displayed an increased sensitivity to hydrogen peroxide and UV radiation, and when trying to repair these lesions they appeared to be less effective than the same cell type from non-AMD patients, suggesting that cellular response to mitochondrial and nuclear DNA damage may be involved in AMD pathogenesis. This work also demonstrated that mitochondrial DNA accumulates more DNA lesions than nuclear DNA in AMD. Synowiec et al. [31] described the association between polymorphisms of the NQO1, NOS3, and NFE2L2 genes and AMD. Other mitochondrial DNA H and J polymorphisms associated with protection or risk for AMD have been described by other authors [32] in a case control study. It was found that mitochondrial DNA haplogroups confer differences in risk for age-related macular degeneration.

Potential blood biomarkers of OS have extensively been described through the literature, classically involving the malondialdehyde (MDA) by product of lipid peroxidation and the 8-hydroxy-2′-deoxyguanosine, a metabolite of nucleic acid oxidation [33]. Several molecules have also been reported as probable biomarker candidates for the early diagnosis of OS in AMD [34, 35]. To explore the proteomic profile of the aqueous humor from wet AMD patients Yao et al. [36] analysed samples from wet AMD and non-AMD patients describing a significantly different protein composition between them. The specific proteins that were identified by the authors, such as galectin 3 binding protein, fibronectin, clusterin, matrix metalloproteinase-2, and pigment epithelium derived factor, may be considered as presumptive markers of wet AMD development. ROS are also produced by endoplasmic reticulum stress. Folding and secretion of proteins produce ROS and endoplasmic reticulum stress has also been described as a primary pathogenic mechanism leading to AMD [37].

It has also been shown that the iron overloaded retina is a useful model for degeneration studies. In this context Rodríguez Diez et al. [38] reported that the OS induced in the retina in the presence of iron seems to be related to AMD by means of cytosolic phospholipase A2 (cPLA2) and calcium-independent isoform (iPLA2) actions. Specifically the Group V secretory PLA2 (sPLA2), a member of PLA2 family, has several intracellular targets during iron-induced retinal degeneration, and its role could be related to inflammatory responses by its participation in cyclooxygenase (COX)-2 and nuclear factor kappa B (NF-*κ*B) regulation.

Further research with large clinical and epidemiological studies as well as animal models and “*in vitro*” experiments are urgently needed to better understand the relationship between OS and AMD.

### 2.2. Angiogenesis

**Figure 2:**
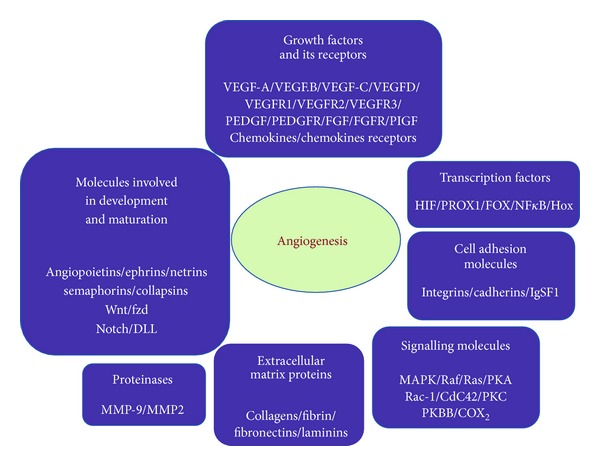
Molecules involved in angiogenesis. There are many molecules that mediate-regulate angiogenesis. The seven classes enclosed in the figure are (1) growth factors and its receptors, (2) transcription factors, (3) cell adhesion molecules, (4) signalling molecules, (5) extracellular matrix proteins, (6) proteinases, and (7) molecules involved in development and maturation. Several of these molecules have been considered for potential diagnosis or therapeutic approaches to control pathological angiogenesis.

Angiogenesis is involved in many diseases, including wet AMD [39, 40]. Key regulators of angiogenesis are the vascular endothelial growth factor (VEGF), pigment epithelium-derived growth factor (PEDGF), fibroblast growth factor 2 (FGF2), angiopoietins, and extracellular matrix molecules (Figure 2). However, VEGF is really the molecular switch for a wide variety of neovascular conditions occurring in the eyes, through its actions on the proliferation and survival of endothelial cells and vascular permeability [41, 42].

Endothelial cells are particularly susceptible to exogenous and circulatory agents. It is evident that the vasculature of different organs responds differently to cytokines and growth factors. Vascular endothelial growth factor (VEGF) activates endothelial cell growth and induces angiogenesis [43–45]. Among the growth factors involved in the exudative AMD, VEGF has been shown to be a major contributor to angiogenesis, by causing a massive signalling cascade in the capillaries of the retina and choroid [46].

Bhutto et al. [47] examined the localization and levels of the VEGF and PEDGF (an antiangiogenic factor) in aged human choroid for determining if the localization or their levels changed in AMD eyes. Data from this study suggest a critical balance between PEDGF and VEGF. In addition, PEDGF may counteract the angiogenic potential of VEGF. The authors concluded that a decrease in PEDGF may disrupt the balance by favouring the formation of choroidal neovascularization in AMD.

Stefater et al. [48] reported a new pathway through myeloid cells that utilize the integration/Wingless (Wnt) pathway to regulate expression of human receptor-type tyrosine kinase gene (Flt1) and angiogenesis (Figure 2). This gene encodes a protein called VEGF receptor-1 that inhibits vascular growth by binding VEGF. It seems that Flt1 expression can also be regulated so that when increasing it blocks VEGF and vascular branching, or when lowering it permits VEGF to increase neovascularization.

It is evident that through VEGF research it has been possible to design new therapeutic strategies for the angiogenic eye disorders, as in AMD [49]. Anti-VEGF agents are currently clinically available for ocular neovascularization treatment, via intravitreal injections.

### 2.3. Apoptosis

Apoptosis is the programmed cell death, the cellular suicide that is essential for the normal development and survival of many organisms. Apoptosis can be classified into three distinct functional steps: induction, effector, and execution. At this moment, the affected cell undergoes an irreversible degradation of all organelle and membranes [50]. From a molecular viewpoint mitochondrial membrane increases permeability and induces the release into the cytosol of proapoptotic factors such as procaspases, caspase activators, and other caspase-independent factors such as the apoptosis-inducing factor, leading to cell death [51].

Experimental studies increasingly provide support that OS and apoptosis are closely linked processes and that both are implicated in the pathophysiologic mechanisms of a wide spectrum of chronic and degenerative disorders. Evidence suggests that ROS result in apoptosis of RGCs and progressive vision loss in primary open-angle glaucoma [34], as well as in AMD eyes [50–52]. Apoptosis has specifically been involved in the early outgrowth of choroidal neovascular membranes, as well as during development of fibrotic scars at later AMD stages [39, 51].

Recent studies demonstrated a significant increase in the terminal deoxynucleotidyl transferase dUTP nick end labelling (TUNEL)-positive cells through the retinal layering of postmortem human eyes with AMD. Moreover photoreceptors in these AMD eyes upregulate Fas, a potential mediator of apoptosis, suggesting that Fas/FasL may trigger the initiation of photoreceptor apoptosis in AMD [51].

**Figure 3:**
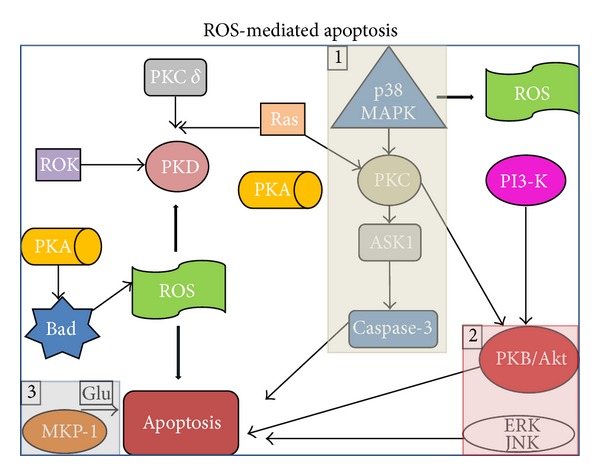
ROS-mediated apoptosis. In order to transmit physiological ROS-mediated signals and to accommodate to the OS, the cells have a wide spectrum of intracellular signal transduction systems, including protein kinase cascades. Much of these pathways are engaged in the route to apoptosis, including MAP kinases/ASK1 upstream regulator, PKB/Akt-ERK. MKP/1 and glutamate-induced cell death. Abbreviations: Akt protein kinase B (PKB), a serine/threonine-specific protein kinase; ASK1 apoptosis signal-regulating kinase 1, ERK extracellular signal-regulated kinase, Glu glutamate, JNK c-Jun NH2-terminal kinase, MAPK mitogen-activated protein kinase, MKP-1 MAPK phosphatase-1, PI3-K phosphatidylinositol 3-kinase, PKA protein kinase A, PKB protein kinase B, PKC protein kinase C, PKD protein kinase D, ROK Rho kinase, and ROS reactive oxygen species.

In this scientific background it has been demonstrated that antioxidants and free radical scavengers, as well as the overexpression of the antioxidant enzyme manganese superoxide dismutase, can inhibit or delay apoptosis [53–55]. Moreover, the B cell lymphoma 2 (Bcl-2) protein has been proved to prevent cells from apoptotic death by means of an antioxidant mechanism [56]. Therefore, it has been suggested that ROS itself, and the resulting cellular redox change, can be part of the signal transduction pathways involved in apoptosis [57], as shown in Figure 3.

### 2.4. Inflammation

**Figure 4:**
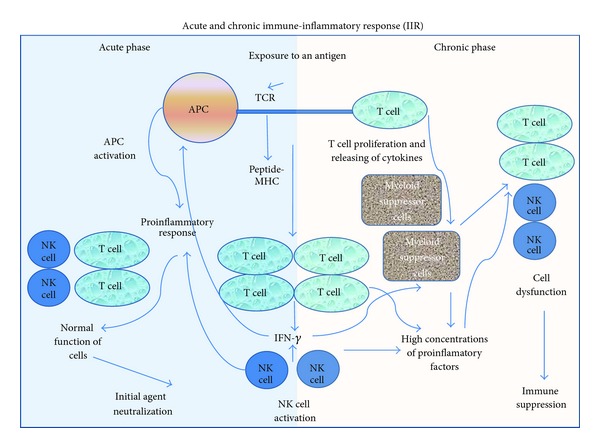
Inflammation and immune response (IIR). The primary defense mechanism of the immune system needs the activation of different cell phenotypes and intercellular signals to orchestrate all actions. Among the cytokines, the interleukines (IL) IL-1 and IL-6 and the TNFa are inducers of the IIR through the regulation of the monocytes. Immunocompetent cells are essential for an adequate immune system function, such as the macrophages, neutrophils, fibroblasts, and endothelial cells.

Immune-inflammatory response (IIR) attempts to rescue the organism from the cell injury and its related effects. IIR involves leukocytes/other innate immune cells and lymphocytes T, B, and NK (adaptive immunity), interacting among them by cytokines, chemokines, nitric oxide (NO), and so on [58]. Tissue damage results from uncontrolled chronic inflammation (Figure 4).

Increased inflammatory plasmatic markers such as the tumor necrosis factor-alpha, TNF alpha, sVCAM-1, E-selectin, interleukins-6 and -18, and MCP-1 have been shown to positively correlate with age, independently of any other cardiovascular risk factors [59]. High levels of inflammatory/immune mediator molecules contribute to a proinflammatory environment that helps to develop vascular dysfunction and promotes endothelial apoptosis in aging, as suggested by Ungvari et al. in a recent review [57].

Drusen constitutes a characteristic change occurring in the macula in relation to aging and is considered an early sign of AMD. Although many authors have investigated the composition and characteristics of human drusen, further research is needed to elucidate its significance for AMD prevention. A recent review by Parmeggiani et al. [60] indicated the pathogenic role of immunologic processes in AMD occurrence, consisting of production of inflammatory related molecules, recruitment of macrophages, complement activation, microglial activation, and accumulation within the macula. This report and other similar reports also emphasized that proteins associated with inflammation and immune response are prevalent among drusen constituents [61–63]. The authors especially focused on the importance of these new findings to improve managing of AMD patients and to prevent severe vision loss.

Altered cytokine profiles of human retinal pigment epithelium have also been related to the aging eye and AMD [34, 64]. Moreover, oxidant injury and replicative senescence infiltration of proinflammatory m1 macrophages have been detected in the outer retina and precede damage in a mouse model of age-related macular degeneration [63].

Pathogenic mechanisms of AMD related to inflammation include variations in complement factor, a mayor risk factor to photo-oxidative stress and inflammation, as well as matrix metalloproteinases activity (recently described in relation to wet AMD and pro-angiogenic environment). In fact, the administration of soluble complement inhibitor Crry-Ig reduces inflammation [65].

Further research in the topic of inflammation and immune response is needed to better understand AMD and to prevent AMD-related visual disability.

## 3. Expanding Our View with Studies on the Role of Antioxidants and Omega-3 Fatty Acids in AMD

### 3.1. Controlled Clinical Trials

Table 1 summarizes the main publications on the effects of antioxidants and/or omega 3 PUFAS for AMD.

#### 3.1.1. With Positive Effects****

*(1) Antioxidants.* Several randomized clinical trials (RCTs) evaluated the effects of antioxidant supplements in patients with Categories 2 to 4 AMD [6, 66–70]. Some of them examined a mixture of antioxidant nutrients, including vitamin C, vitamin E, and some carotenoids such as beta-carotene or quercetin [67–70]. Other studies used zinc alone in the form of zinc sulfate (200 mg/d) [66, 68] or as zinc monocysteine (50 mg/d) [69].

By far, the largest study was the multicenter AREDS trial which randomized 3640 patients with Categories 2, 3, and 4 AMD into four treatment groups: antioxidants with carotenoids, zinc alone, antioxidants with carotenoids + zinc, or placebo [6]. The primary outcome was progression to advanced AMD (central geographic atrophy or choroidal neovascularization) and at least moderate functional visual loss, defined as the loss of ≥15 letters on the ETDRS logMAR chart. A secondary visual outcome was a decrease in the best corrected visual acuity score from baseline of 30 or more letters in a study eye (six lines or a quadrupling of the initial visual angle) and progression of disease to a visual acuity score worse than 20/100 in one or both eyes. Overall, in analyses limited to only those with Category 3 or 4 AMD, a reduction in functional visual loss was noted with either supplement alone or in combination. Specifically, after five years of follow-up, the zinc/antioxidant combination significantly decreased the degree of functional vision loss (OR 0.63; 99% CI 0.44–0.92). A similar but smaller effect was noted with either supplement alone (zinc alone OR 0.75; 99% CI 0.53–1.07; antioxidants alone OR 0.79; 99% CI 0.55–1.13). On the other hand, antioxidants were not associated with benefit in the large subgroup of patients with Category 2 AMD (*n* = 1063) over seven years of follow-* *up. Of note, there was a very low rate of progression from Category 2 AMD to more advanced disease. Of patients with Category 2 AMD at baseline, 13 progressed to advanced AMD and 316 progressed to Category 3 or 4 AMD during the study. No significant differences in demographics, socioeconomic status, smoking status, or comorbidities were noted between Category 2, 3, or 4 participants.

One additional trial found benefit from supplement use [66]. In this small trial, 90 patients with Category 3 or 4 AMD were randomized to either zinc sulfate supplements (200 mg/day) or placebo. Seventy-one subjects in the placebo and 80 subjects in the zinc group completed the two-year follow-up. Most patients had Category 3 or 4 AMD at baseline, and half the participants had baseline evidence of geographic atrophy. The placebo group was twice as likely as the zinc- supplemented group to demonstrate clinically significant vision loss (15.5% versus 7.5%).

Other studies were small (*n* = 56 or less), of relatively short duration (6 to 24 months), and did not report a clinically significant reduction in visual loss from supplement use. [67–69]. Two of these trials were conducted in VA settings.

One randomized trial reported the effects of a supplemental carotenoid, lutein (10 mg/d), among subjects with AMD [67]. Subjects were randomized to lutein alone (*n* = 29), lutein + antioxidants (OcuPower; *n* = 30), or placebo (*n* = 31) for 12 months. The authors report very small increases in visual acuity in the lutein and lutein + antioxidants groups, but these changes were not clinically significant (<15 letters) after a one-year follow-up.

Another small, multicenter VA trial evaluated the effects of a tablet containing a number of antioxidants and, similarly, found nonclinically significant improvement in visual acuity [70].

*(2) Omega-3 Fatty Acids*. One study examined the effects of supplemental omega-3 fatty acids, L-carnitine, and coenzyme Q10 among subjects with AMD [71]. Most (93.6%) subjects in this study had “early” AMD at baseline; only 6.4 percent had Category 3 or 4 AMD. The study randomized subjects to omega-3 fatty acid supplement (*n* = 52) or placebo (*n* = 55). The change in visual acuity was measured after 12 months of supplementation. The report noted that omega-3 fatty acids slowed visual acuity loss but not to a clinically significant degree (0.5 line change in Snellen acuity). Other studies analysed the role of dietary omega-3 PUFAS and fish intake in the primary prevention of AMD [72]. Christen et al. [73] in a large cohort of female health professionals who were free of a diagnosis of AMD at baseline, examined the relation of dietary intake of DHA/EPA and fish with visually significant AMD during 10 years of follow-up. Results from this study demonstrated that regular consumption of DHA and EPA and fish was associated with a significantly decreased risk of incident AMD. Authors suggested that this intervention may benefit primary prevention of AMD. 

*AREDS2.* The National Institute of Health (NIH) commissioned the National Eye Institute to conduct the Age-Related Eye Disease Study 2 (AREDS2) [7]. It was a multicentered, randomized, double-masked, placebo-controlled phase 3 study with a 2 × 2 factorial design, conducted in 2006 to 2012 and enrolling 4203 participants aged 50 to 85 years at risk of progression to advanced AMD with bilateral large drusen or large drusen in one eye and advanced AMD in the fellow eye. The purpose of the study was to determine whether adding lutein + zeaxanthin, DHA + EPA, or both to the AREDS formulation decreases the risk of developing advanced AMD. Additionally, the treatment arms evaluated the effects of eliminating beta-carotene from the original AREDS formulation of antioxidants (500 mg vitamin C; 400 IU vitamin E; and 15 mg beta-carotene) and/or lowering the zinc component of the supplement (previously 80 mg zinc as zinc oxide, and 2 mg copper as cupric oxide). Participants were randomized to receive lutein (10 mg) + zeaxanthin (2 mg), DHA (350 mg) + EPA (650 mg), lutein + zeaxanthin and DHA + EPA, or placebo. All participants were also asked to take the original AREDS formulation or accept a secondary randomization to 4 variations of the AREDS formulation, including elimination of beta-carotene, lowering of zinc dose, or both. After a median follow-up of 5 years, 1940 study eyes (1608 participants) progressed to advanced AMD. Kaplan-Meier probabilities of progression to advanced AMD by 5 years were 31% for placebo, 29% for lutein + zeaxanthin, 31% for DHA + EPA, and 30% for lutein + zeaxanthin and DHA + EPA. Comparison with placebo in the primary analyses demonstrated no statistically significant reduction in the progression to advanced AMD (hazard ratio [HR], 0.90 [98.7% CI, 0.76–1.07]; *P* = .12 for lutein + zeaxanthin; 0.97 [98.7% CI, 0.82–1.16]; *P* = .70 for DHA + EPA; 0.89 [98.7% CI, 0.75–1.06]; *P* = .10 for lutein + zeaxanthin and DHA + EPA). There was no apparent effect of beta-carotene elimination or lower-dose zinc on progression to advanced AMD. More lung cancers were noted in the beta-carotene versus no beta-carotene group [2.0%] versus 11 [0.9%], *P* = .04, mostly in former smokers.

The study concluded that addition of lutein + zeaxanthin, DHA + EPA, or both to the original AREDS formulation in primary analyses did not further reduce risk of progression to advanced AMD. However, because of potential increased incidence of lung cancer in former smokers, lutein + zeaxanthin could be an appropriate carotenoid substitute in the AREDS formulation.

#### 3.1.2. Without Effects

*(1) Antioxidants.* Antioxidants have been hypothesised to reduce oxidative damage to the retina, but the effectiveness of dietary oxidants in the primary prevention of AMD is unclear.

Previous studies and reviews have largely focused on the role of dietary antioxidants and supplements in the prevention of early AMD or progression to late AMD in people with signs of early disease. Among all the clinical trials done, most of them are prospective cohort studies. Only four are randomized clinical trials (including the AREDS2, recently released).

All the studies dealing with the role of antioxidants and supplements found that there is little or no effect of vitamins (A, C, and E) zinc, lutein, zeaxanthin, other carotenoids, and omega-3 fatty acids [73] to prevent AMD.

*Dietary Antioxidants and Early AMD.* Vitamin A: the studies that contributed to the pooled analysis reported null associations between intake of vitamin A and prevention of AMD [74–76].

Vitamin C: due to the heterogeneity of the studies, there are reported positive associations [77] as well as inverse [76] or null effect in preventing AMD [74–76].

Vitamin E: most of the cohort and randomized studies published have reported null or inverse association between intake and preventing AMD [74, 77, 78]. In randomized trials, no protective effect was seen for vitamin E.

Zinc: results are contradictory. Most of them reported positive associations except one null [77] and other inverse association [74].

Lutein and zeaxanthin: none of the findings in the studies were statistically significant, most of them showed null [78, 79] or even inverse correlation between their supplementation and prevention of progression of AMD [76]. The AREDS2 [7] found no benefit in the addition of lutein plus zeaxanthin to the AREDS formulation to reduce risk of AMD progression.

Omega-3 fatty acids: although few prospective studies and meta-analysis suggest that the consumption of fish and foods rich in omega-3 fatty acids may be associated with low risk of AMD, there is insufficient evidence to support their routine consumption for AMD prevention [80–82]. Only one randomized clinical trial (AREDS2) concluded that DHA plus EPA added to the AREDS formulation did not further reduce the risk of progression to advanced AMD [78].

*Dietary Antioxidants and Late AMD.* Only few published cohort studies provided data for risk of late AMD. Each of them evaluated different antioxidants. There is insufficient evidence that antioxidants at the studied doses prevent the progression to late AMD [83, 84].

#### 3.1.3. With Adverse Effects

Treatment options for nonneovascular Age Macular Degeneration (AMD) are limited and no prophylaxis is still available. Several randomized clinical trials (RCTs) have been done during the last two decades based on the beneficial effects of antioxidants, vitamins, and zinc. The benefits of these micronutrients have been demonstrated but although generally reported as safe, vitamin supplements may also have harmful effects.

More than thirty large RCTs with more than one year of follow-up have been registered to study the efficacy and safety of oral supplements. The evidence for harms has been driven by two trials, the Alpha-Tocopherol, Beta-Carotene (ATBC) Lung Cancer Prevention Study, and the Carotene and Retinol Efficacy Trial (CARET) [84–87]. Vitamin E at high doses seemed to be associated with increased risk of mortality, congestive heart failure, prostate cancer, and beta-carotene with an increased risk of lung cancer among active smokers (Table 2).

The Age-Related Eye Disease Study (AREDS) [6], a multicentered, prospective, randomized trial sponsored by the National Eye Institute of Health, was designed to assess the clinical course of AMD and to evaluate the effect of high doses of vitamin C, vitamin E, beta-carotene, and zinc on the progression of this disease. In 2001 the results were published [6]. From the efficacy point of view a reduced risk of advanced age-related macular degeneration and vision loss for study participants with some degree of AMD who were assigned to high-dose supplementation with antioxidants plus zinc was reported. Regarding safety, circulatory adverse effects were more frequently found in the zinc groups (*P* = .01) as well as genitourinary hospitalizations (*P* = .001) and hospitalizations for mild/moderate symptoms (*P* = .04). Skin and subcutaneous tissue conditions (yellow skin) were more frequent in the antioxidant arms (*P* = .003). Although the risk of lung cancer among smokers was not established in this study, the data and safety monitoring committee recommended that smokers should discontinue study medications containing beta-carotene based on the results from the RTCs that had suggested increased risk of mortality among smokers supplemented with beta-carotene [84–86].

Beta-carotene intake also seems to predict neovascular AMD in both smoker and nonsmokers and higher intakes of total vitamin E predict late AMD as it was shown in the Blue Mountain study [87]. On the other side, several studies with lutein, zeaxanthin, vitamins, and omega-3 fatty acids have been reported to decrease AMD progression [88] Omega-3 long-chain polyunsaturated fatty acids have been recently mentioned to have a potential benefit in patients with AMD or those at risk of AMD, and although several studies have demonstrated that high dietary intake of omega-3 fatty acids is associated with a 38% decreased risk for late AMD, there are not many RCTs with this micronutrient [71–73].

The AREDS 2 [7], a multicentered, randomized, controlled phase 3 clinical trial with a 2 × 2 factorial design, conducted between 2006 and 2012 and enrolling 4203 participants aged 50 to 85 years at risk of progression to advanced AMD was designed to determine whether adding lutein + zeaxanthin, DHA + EPA, or both to the initial AREDS formulation decreases the risk of developing advanced AMD. The results have been recently published. The addition of lutein + zeaxanthin, DHA + EPA, or both to the AREDS formulation do not reduce risk of progression to advanced AMD and regarding safety, more lung cancers were noted in the beta-carotene versus no beta-carotene group (2.0% versus 11 0.9%, *P* = .04), mostly in former smokers [7]. It is known that the elevated zinc concentration used in AREDS causes minor side effects, such as stomach problems. Copper, as cupric oxide, was added to avoid anemia induced by high zinc intake. Moreover, it was stated from ARED2 results that while zinc is an important component of the formulation, it is now unclear how much zinc is necessary for the purpose of the study.

With the megadoses of vitamins and minerals that have been utilized by different studies, it has to be taken into consideration that secondary effects can appear. In fact, vitamin E appropriate dosage can be confusing. However, current guidelines [89] instructed about dietary allowances (RDA) and upper tolerable threshold for vitamins. But, it is usually seen that most commercial products remain labeled in International Units (IUs), instead of mg, as recommended. Vitamin E supplementation may potentiate the effects of Warfarin (Coumadin). Therefore, taking vitamin E along with the latter can increase the chances of bleeding. Vitamin E supplementation has been associated with increased risk of heart failure. Taking into account all the above information, summarized in Table 1, and although valuable clinical effects of nutritional supplements have been reported, more information about safety in the general population is needed.

### 3.2. Animal Models for AMD and the Effects of Antioxidants Omega-3 PUFA

As shown above some clinical trials have suggested that omega-3 polyunsaturated fatty acids (omega-3 PUFA) decrease the likelihood of developing AMD. Some animal models have also been developed in order to check this hypothesis, mainly in rodents and primates (Table 3).

Mammals depend on dietary intake of omega-3 PUFA, because mammalian cells lack enzymes necessary to synthesize the precursor of omega-3 PUFA and to convert omega-6 to omega-3 PUFA [86, 90]. Sufficient omega-3 PUFA are principally derived from fish and seafood and are difficult to obtain from purely vegetarian sources. The animal models have been tested with high or low dietary intake of omega-3 PUFA.

On the one hand, the use of murine models can provide basic physiology and pathology relevant to human AMD, because they are relatively cheap and easily reproduced and manipulated. However, rodents have no macula and their retinal photoreceptor cells are predominantly rods, rather than the cone cells that predominate in the human macula. What cones the rats do have are dispersed throughout the retina, rather than concentrated around the fovea as they are in humans [91].

In one study, four-week supplementation with 5% EPA was shown to prevent choroidal neovascularization induced after laser photocoagulation in 6-week-old C57BL/6 mice [92]. EPA also exerted anti-inflammatory action locally in the eye, as well as systemically in the circulation by means of significant reduction in the expression and production of inflammatory and angiogenic markers in the RPE, endothelial cells, and macrophages, and lower serum CRP and IL-6.

Cx3cr1 and Ccl2-deficient mice (double-knockout) have been shown to present a synergistic effect resulting in a phenotype displaying typical AMD features with early onset and high penetrance [93, 94]. A diet enriched in DHA and EPA for 12 weeks in 9-week-old Ccl2/Cx3cr1 deficient mice can ameliorate the progression of retinal lesions such as retinal and subretinal spots, chorioretinal scars, focal loss of photoreceptors, and focal hypopigmentation of the RPE cells. The authors proposed the increase of anti-inflammatory derivatives such as PGD2 and the decrease of proinflammatory derivatives such as PGE2, LTB2, TNF-alpha, and IL-6 as the mechanisms underlying the lower disease progression [94, 95].

On the other hand, nonhuman primates offer the closest anatomy to humans, but are quite difficult to manipulate genetically, costly to maintain, have a slow time course of disease progression, and some researchers are also loath to conduct research in primates for ethical reasons [90, 91]. Macaque monkeys have a macula and commonly develop age-related maculopathy that shares common features and risk factors with human AMD. Monkeys rarely show spontaneous development of either the wet or dry form of advanced AMD, but it has been documented the signs of early to intermediate AMD in older rhesus macaque monkeys (Macaca mulatta) [96, 97].

However, there is one monkey model of cynomolgus macques (Macaca fascicularis) that presents an early-onset macular degeneration syndrome [98, 99]. Besides, a similar syndrome has been identified in the Japanese macaque (Macaca fuscata) [100].

Human and monkey drusen has been shown to be histopathologically similar [101]. Furthermore, it appears that both species present similar key nutritional risk factors for AMD development. Monkey-fed-diets lacking lutein-zeaxanthin and omega-3 PUFA showed an increased incidence of drusen at early ages, by 15 years of age (equivalent to 45 human years), and atrophic macular disease [99, 101, 102]. Moreover, adequate intake of omega-3 PUFA reduces the blue-light-induced damage in the parafovea of rhesus monkeys [103]. A summary of these data is enclosed in Table 2.

All these findings provide support for the role of omega-3 PUFA as an important factor in the development of AMD in animal models.

### 3.3. *In Vitro* Models for AMD and Evaluation of Antioxidants and Omega-3 Fatty Acids

Due to its high complexity it is not easy to find *in vitro* models that mimic AMD, mainly for three reasons: the first one and the most important is due to the unawareness of the etiology and physiology of AMD; the second one is as a matter of the complexity of the disease involving a variety of cell types; and the last one but not the least refers to the variety of disease types and findings (exudative AMD, atrophy, drusen, and pigment abnormalities).

#### 3.3.1. *In Vitro* Models of Angiogenesis

Choroidal neovascularization and VEGF play an essential role on exudative AMD.

Endothelial primary cultures are the most useful cell types used for this purpose, as they are the main constituent of blood vessels and its proliferation and migration induced by VEGF play an essential role in the pathologic angiogenesis.

Studies using bovine carotid endothelial cells are described [103], in which the effects of EPA on VEGF-induced MAP kinase activation are tested, as well as the expression of VEGF receptors. The use of primary culture of human umbilical vein endothelial cells (HUVECs) is also considered as a good model for the understanding of the pathology [104]. A study evaluating the antiangiogenic properties of EPA on proliferation, migration, and tube formation of HUVEC cultures has been published with promising results [105]. The use of human aortic endothelial cells [105] is another alternative. Finally, the most useful cell type for the study and the understanding of proliferative eye pathologies might be the retinal microvascular endothelial cells, mostly extracted from bovine [106] or porcine [107] eyes, although PUFAs antiangiogenic properties have not been tested yet on it.

#### 3.3.2. *In Vitro* Models of Oxidative Stress

Although the mechanisms involved in AMD are still not completely understood, it is well known that oxidative stress plays a major role in the evolution of this pathology. Cells undergoing oxidative stress produce proinflammatory and proangiogenic factors and they are more susceptible to apoptosis.

The classical way to produce oxidative stress is the reduction of the oxygen concentration in the environment. The hypoxia/reoxygenation model in HUVEC cultures [108] shows an increased production of reactive oxygen species and a dysfunction in gap junctions, which is reversed by EPA pretreatment.

Another good approach to mimic the oxidative stress produced in the retina is the use of oxidant agents. One of the most extended methods is the addition of H_2_O_2_ in the cultures. This approach has been used to demonstrate the protective effect of DHA derivative neuroprotectin D1 in ARPE-19 cells (a spontaneously transformed human RPE cell line), showing DNA fragmentation reduction, caspase cleavage reduction, and upregulation of antiapoptotic proteins [109]. The difficulty of this technique lies in conveniently diluting H_2_O_2_ to generate stress without causing widespread cell death. Other oxidant agents can also be used to generate stress, for example, paraquad [110]. In this study they isolate neuronal cultures from rat retinas and expose them to the oxidant, testing the protective properties of DHA and macular carotenoids. The cell viability, apoptosis, and the mitochondrial membrane potential are evaluated. Finally, tert-butyl hydroperoxide (tBH) has also been demonstrated to cause an oxidative stress *in vitro* in ARPE-19 cultures [111], and it has been used in the study of the efficacy of various antioxidants protecting the retinal pigment epithelium from oxidative stress [112–114], although PUFAs protection has not been tested in this model yet.

The last model of oxidative stress is the light stress model [115] based on *in vitro* liposomal membranes reproduction mimicking the macular membranes of human retina, and posterior UV radiation to produce oxidative damage. This model has only been used to test the effect of lutein and zeaxanthin on lipid oxidation, but this might be also a promising tool for the research of antioxidant properties of PUFAs.

## 4. Conclusions and Future Directions

The AMD is a leading promoter of visual disability in people over their sixties, with an exponentially growing prevalence that continues to rise. The AMD pathogenesis involves a complex interaction of cellular and molecular factors, which may be induced by light damage, OS, angiogenesis, apoptosis, and/or inflammation. In this context, it is evident that nutrients and other dietary components may contribute to AMD. Although new treatments for AMD have appeared in the past years, which have notably improved visual outcomes and quality of life, disappointing clinical results are still present in the clinical practice. Therefore, knowledge on the pathogenic mechanisms of AMD remains far from complete. With a simple view to reduce OS, apoptosis, inflammation, and/or angiogenesis, the role of nutritional antioxidant supplements and omega-3 PUFAs on the initiation and progression of AMD are currently unresolved. However, wide circumstantial evidences from epidemiological studies and preclinical “*in vivo*” and “*in vitro*” research are strongly supportive.

Many observational studies have suggested a benefit from high dietary intakes of omega-3 and macular pigments like lutein and zeaxanthin which are associated with a lower risk of prevalence and incidence in AMD. However, in the case of omega-3 more clinical trials are required for concrete recommendations on their use. The Age-Related Eye Disease study (AREDS) showed a beneficial effect of high doses of vitamins C, E, beta-carotene, and zinc with copper in reducing the risk of progression to advanced AMD in patients with intermediate AMD or in patients with one-sided late AMD. The AREDS-2 study has shown that lutein and zeaxanthin may substitute beta-carotene because of potential increased incidence of lung cancer. For summarizing the effects of antioxidants and w-3 fatty acids see Table 1.

In Europe, the doses of vitamins and minerals used in addition to lutein and omega-3 are typically lower than the doses used in the two AREDS studies and in other larger observational studies. In addition, dietary habits, environmental factors, medical conditions, lifestyles, and genetic background are different in the European countries from the population involved in the AREDS. In that scenario, there are no data about the protective effect of these nutritional supplements on the secondary prevention of AMD. So, European-based study would be very helpful to determine the effect of nutritional supplements in AMD prevention.

In the near future new devices and new designs will improve the diagnostic and therapeutic options for AMD at different stages with the challenge of personalizing the patient management and standards of care.

## Figures and Tables

**Table 1:** Publications on the effects of antioxidants and/or omega 3 polyunsaturated fatty acids supplements for AMD.

Evaluation	Antioxidants	Omega 3 fatty acids
With positive effects	AREDS report 8; Arch Ophthalmol 2001 (see [6]) AREDS2 RCT. JAMA 2005 (see [7]) Newsome et al., Arch Ophthalmol 1988 (see [66]) Richer et al., Optometry 2004 (see [67]) Stur et al., IOVS 1996 (see [68]) Newsome DA. Current Eye Res 2008 (see [69]) Richer S. J Am Optom Assoc 1996 (see [70])	Feher et al., Ophthalmologica 2005 (see [71]) Chong et al., Arch Ophthalmol 2008 (see [72]) Christen et al., Arch Ophthalmol 2011 (see [73])

Without effects	AREDS2 RCT. JAMA 2005 (see [7]) Leeuwen et al., JAMA 2005 (see [74]) Flood et al., Ophthalmology 2002 (see [75]) Chow et al., Arch Ophthalmol 2004 (see [76]) Van den Langenberg et al., Am J Epidemiol 1998 (see [77]) Taylor et al., BMJ 2002 (see [79]) Moeller et al., Arch Ophthalmol 2006 (see [78]) Teikari et al., Acta Ophthalmol Scand. 1998 (see [83]) Omenn et al., N Engl J Med 1996 (see [86])	AREDS2 RCT. JAMA 2005 (see [7]) Hodge et al., Ophthalmol 2006 (see [80]) Chong et al., BMJ 2007 (see [81]) Soused et al., Ophthalmology 2013 (see [82]) Heinonen et al., Annals of Epidemiology 2004 (see [84]) Tan et al., Ophthalmology 2008 (see [87]) Olson et al., Semin Ophthalmol 2011 (see [88])

With negative effects	AREDS report 8; Arch Ophthalmol 2001 (see [6]) AREDS2 RCT. JAMA. 2013 (see [7]) The ATBC Cancer Prevention Study Group. Ann Epidemiol 1994 (see [84]) Goodman GE et al., Cancer Epidemiol Biomarkers Prev 1993 (see [85]) Omenn et al., N Engl J Med 1996 (see [86])	AREDS2 RCT. JAMA 2005 (see [7])

**Table 2:** RCTs: Beta-carotene and risk of lung cancer in smokers.

Randomized clinical trials (RCTs)	*N *	Treatment groups	Follow-up	Lung cancer risk (smokers)	*P* value
The Alpha-Tocopherol, Beta-Carotene (ATBC) Lung Cancer Prevention Study—1985–1993 (see [84])	29133 smokers (men)	Group 1: *α*-tocoferol Group 2: *β*-carotene Group 3: Placebo	5–8 years	Beta-Carotene supplementation was associated with increased lung cancer risk (RR = 1.16; 95% CI = 1.02–1.33)	*P* = .02

The carotene and retinol efficacy trial (CARET)-1993 (see [85])	18314 smokers (men and women) or asbestos exposed	Group 1: *β*-carotene and retinyl palmitate Group 2: Placebo	*Stopped 21 months earlier *	There were 28% more lung cancers and 17% more deaths in the active intervention group	*P* = .01

**Table 3:** Summary of different animal models studied in relation to dietary omega-3 PUFA.

Authors	Animal model	Results
Koto T et al., 2007 (see [92])	C57BL/6 mice	Supplementation with EPA prevented choroidal neovascularization induced after laser photocoagulation

Chan et al., 2008 (see [94])	*Cx3cr1* and *Ccl2*-deficient mice	Smaller number of retinal lesions when diet high in omega-3 PUFA

Tuo et al., 2009 (see [93])	*Cx3cr1* and *Ccl2*-deficient mice	Mice that ingested a high omega-3 PUFA diet showed a slower progression of retinal lesions compared with the low omega-3 PUFA group

Neuringer et al., 2010 (see [100]) Renner et al., 2013 (see [102])	Macaque monkeys	Diets lacking carotenoids and omega-3 PUFAs developed patches of RPE atrophy and increased incidence of drusen. SD-OCT showed RPE disruption
